# A hybrid AHP–TOPSIS decision-support framework for evaluating mental health support alternatives in higher education

**DOI:** 10.3389/fpubh.2026.1861300

**Published:** 2026-08-12

**Authors:** Qiongyi Xu

**Affiliations:** School of General Education, Zhengzhou Business University, Zhengzhou, China

**Keywords:** AHP–TOPSIS, blended care model, mental health support, multi-criteria decision-making, university students

## Abstract

**Background:**

Mental health challenges among university students have increased substantially, creating a need for transparent and systematic approaches to support decision-making in the selection of mental health service models. This study proposes an integrated Analytic Hierarchy Process (AHP) and Technique for Order Preference by Similarity to the Ideal Solution (TOPSIS) framework to evaluate alternative mental health support systems in higher education.

**Methods:**

A publicly available SCL-90 mental health dataset and questionnaire responses from 200 university students were used to identify and operationalize five evaluation criteria. The comparative performance of three intervention alternatives, teletherapy, in-person counseling, and blended care, was assessed through structured evaluation by a five-member expert panel informed by the empirical evidence and the literature. Criteria weights were determined using AHP, and TOPSIS was applied to rank the alternatives. Consistency and sensitivity analyses were performed to evaluate the reliability and robustness of the proposed framework.

**Results:**

Mental Health Need and Expected Effectiveness (0.4847) and User Engagement (0.2268) received the highest priority weights. Within the proposed decision support framework, blended care achieved the highest closeness coefficient (0.979), followed by in-person counseling (0.402) and teletherapy (0.240). The pairwise comparisons demonstrated acceptable consistency (Consistency Ratio = 0.0221), and the ranking remained stable under moderate variations in the criteria weights. The findings reflect the comparative performance of the alternatives against the defined evaluation criteria and weighting structure, rather than direct evidence of clinical effectiveness.

**Conclusion:**

The proposed AHP and TOPSIS framework provides a transparent and robust decision support approach for evaluating university mental health service models. The results indicate that blended care offers the most balanced performance across the selected evaluation criteria within the proposed framework. The study demonstrates the applicability of integrated multi-criteria decision-making methods to support evidence-informed mental health service planning in higher education, while highlighting the importance of combining empirical evidence with structured expert judgment.

## 1 Introduction

Mental health conditions among university students are widely recognized as a major public health concern (1–3). A high prevalence of depression, anxiety, and stress-related disorders has been reported during the transition to higher education, where academic pressure and social changes are experienced simultaneously (4). According to the World Health Organization, a significant proportion of mental health disorders emerge during early adulthood, which highlights the importance of effective support systems within universities (5).

Traditional in-person counseling has been widely used and is regarded as clinically effective, particularly for moderate-to-severe conditions (6). Direct interaction and personalized therapeutic processes are identified as key strengths (7). However, limitations are evident in accessibility, cost, and service capacity. Many students experience barriers, such as long waiting times and perceived stigma (8, 9). In contrast, digital mental health interventions, including teletherapy and online platforms, are increasingly adopted due to their flexibility and scalability. Linardon, Jake, et al. (10) indicated that digital interventions achieve significant reductions in depression and anxiety, particularly for mild to moderate conditions (10). Nevertheless, challenges related to sustained engagement and adherence remain significant limitations (11).

Cuijpers, Pim, et al. (12) provided strong justification for the criteria adopted in this study. Mental Health Need and Expected Effectiveness is consistently identified as the primary outcome in mental health evaluation, as symptom reduction remains the central objective (12). User engagement is emphasized as a critical determinant of intervention success, particularly in digital systems that require continuous interaction (13). Low engagement is frequently associated with reduced effectiveness, which supports its inclusion as a key criterion. Accessibility is recognized as a major advantage of digital interventions, as geographical and structural barriers are reduced (14). However, accessibility alone is not sufficient without adequate engagement and quality of interaction. Cost efficiency is also considered an important factor in university settings, where financial constraints influence service utilization (15). Privacy concerns are frequently reported as barriers to help-seeking behavior, particularly in digital environments where data security is a concern (16, 17).

The alternatives considered in this study reflect the current evolution of mental health service delivery systems. Teletherapy (A1) represents digital intervention models that provide remote access to mental health services. These systems offer high accessibility and flexibility, but limitations are reported in maintaining engagement and therapeutic depth (11). In-person counseling (A2) represents traditional service delivery, where direct interaction enables strong clinical outcomes, particularly for complex conditions (7). However, constraints related to availability and scalability are observed. Blended care models (A3) integrate digital tools with face-to-face support, combining the advantages of both approaches. Recent studies demonstrate that blended interventions achieve improved outcomes by enhancing both accessibility and Mental Health Need and Expected Effectiveness (18). In addition, evidence suggests that students prefer systems that combine digital convenience with human interaction (15), supporting the inclusion of blended care as a distinct and important alternative.

From a methodological perspective, evaluating such alternatives requires a structured approach given the presence of multiple, often conflicting criteria. The Analytic Hierarchy Process is widely used to determine criterion weights based on expert judgment (15, 16), while TOPSIS is used to rank alternatives based on their distance from the ideal solution (19). Recent studies confirm that integrating AHP and TOPSIS improves decision accuracy and reduces subjectivity in complex evaluation problems (20). However, limited research applies this integrated framework using real student-level datasets in the context of mental health systems. Despite existing contributions, several research gaps are identified. Most studies examine digital and traditional systems separately, without providing a unified comparative framework. The integration of empirical student-level data with expert-based weighting methods remains limited. In addition, the simultaneous consideration of multiple criteria, including effectiveness, engagement, accessibility, cost, and privacy, is not sufficiently addressed within a single analytical model. Recent studies have demonstrated the effectiveness of digital mental health interventions, blended care models, and integrated AHP and TOPSIS methodologies; however, their integration into a unified decision support framework for the comparative evaluation of university mental health support alternatives remains limited.

This study addresses these gaps by proposing an integrated AHP–TOPSIS framework that combines interpretation of the publicly available SCL-90 College Students Mental Health Dataset, questionnaire responses collected from 200 university students, and expert panel validation. The public dataset was used to identify and structure the evaluation criteria and decision framework, while the questionnaire responses were used to assess student perceptions of the selected criteria and mental health support alternatives. Expert judgment was subsequently incorporated to validate the criteria and perform the AHP weighting process. It should be noted that the empirical data were used to establish and operationalize the evaluation criteria, whereas the comparative performance of the intervention alternatives was assessed through structured expert judgment, as the alternatives were not directly represented as intervention groups in the available dataset.

The novelty of this research lies in the systematic derivation of criteria from real-world data, the structured weighting of these criteria, and the comparative evaluation of teletherapy (A1), in-person counseling (A2), and blended care (A3) within a single decision-making framework. The study is guided by the following research questions: (1) how student-level data can be transformed into meaningful evaluation criteria, (2) what the relative importance of these criteria in mental health support evaluation is, and (3) which alternative provides the most effective solution based on a multi-criteria decision-making approach. This work contributes to both methodological development and practical decision-making in higher education mental health services.

## 2 Methodology

**Figure 1:**
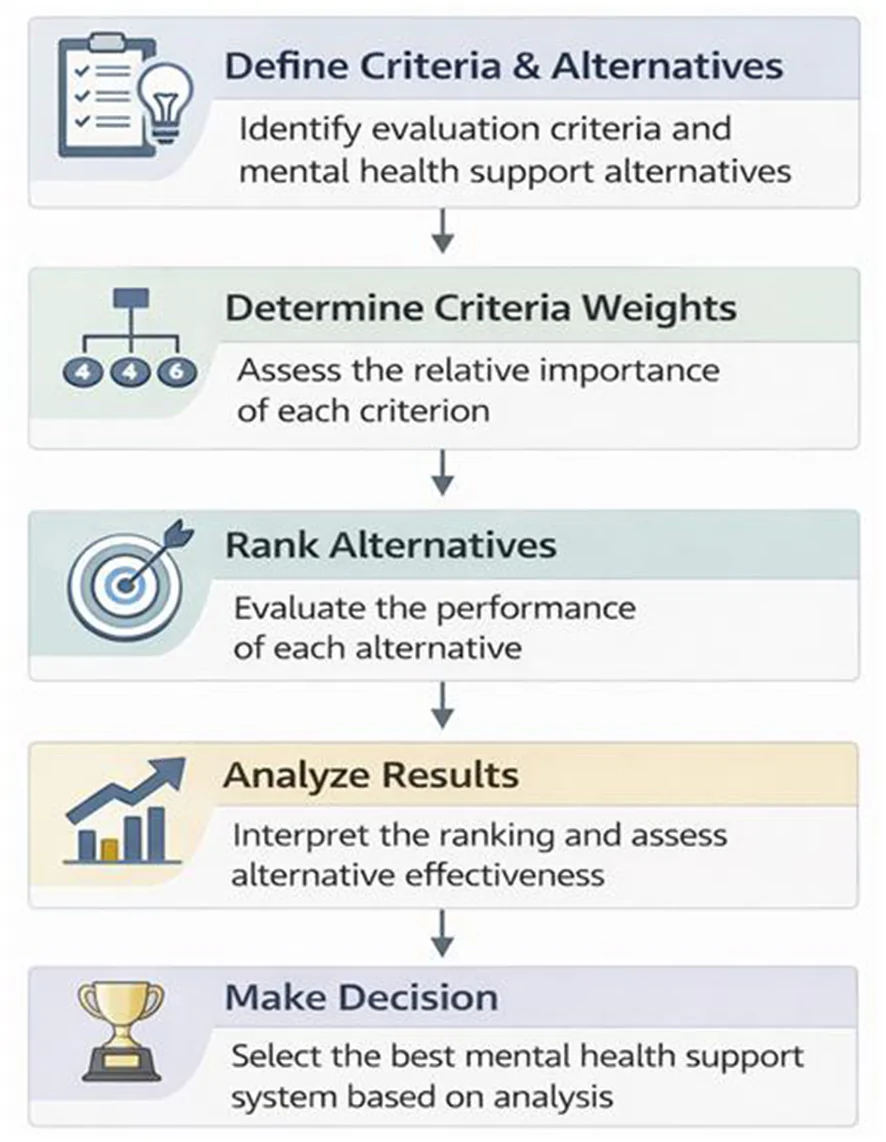
A systematic multi-criteria decision-making framework illustrating the sequential process of data preparation, criteria identification, weighting through pairwise comparison, alternative evaluation, and final decision-making for mental health support systems.

A systematic multi-criteria decision-making framework is adopted to structure the evaluation process. The procedure is organized into a sequence of analytical steps that ensure logical progression from data handling to final decision outcomes. Initially, data are prepared and relevant evaluation criteria are identified to reflect the key dimensions of the assessment. Decision alternatives are then defined to represent different categories of mental health support systems. The relative importance of the criteria is determined using a pairwise comparison approach, and a ranking technique is subsequently applied to evaluate each alternative's performance against the selected criteria. The integration of these methods enables a balanced assessment across multiple dimensions while maintaining methodological consistency. The overall research framework and workflow are presented in Figure 1.

### 2.1 Research design and data collection

A quantitative research design is adopted based on a structured dataset of university students. The dataset is first interpreted to understand its composition and relevance for decision analysis. Each row represents an individual respondent, while the columns include demographic attributes and multiple psychological and behavioral indicators, such as measures of depression, anxiety, somatic symptoms, and other response variables. These variables do not directly represent decision criteria; therefore, an initial interpretation step is conducted to identify how they can inform meaningful evaluation dimensions.

The study employed a combined approach to secondary and primary data. First, the publicly available SCL-90 College Students Mental Health Dataset, obtained from Mendeley Data, was analyzed to identify relevant psychological and behavioral variables and to inform the development of evaluation criteria for the AHP–TOPSIS framework. The public dataset served as the foundation for the construction of criteria and conceptual mapping. Subsequently, a questionnaire survey of 200 university students was conducted to obtain participants' perceptions of the identified evaluation dimensions and mental health support alternatives. The questionnaire responses were not used as a replacement for the public dataset but were incorporated to validate and operationalize the criteria within the decision-making framework.

Before constructing the composite criterion scores, the internal consistency of each multi-item questionnaire construct was evaluated using Cronbach's alpha. Reliability analysis was conducted to verify that the questionnaire items assigned to each evaluation criterion exhibited acceptable internal consistency prior to aggregation. Only constructs demonstrating satisfactory reliability were retained for subsequent aggregation and incorporation into the AHP–TOPSIS framework.

Based on this interpretation, groups of variables are mapped to criteria for evaluating mental health support. Psychological symptom variables are used to reflect Mental Health Need and Expected Effectiveness, while selected response and behavioral indicators are associated with user engagement and accessibility. Cost efficiency and privacy are incorporated conceptually, guided by questionnaire items and expert judgment. This step provides a structured foundation for selecting criteria in the subsequent AHP analysis, ensuring that the dataset is not used in its raw form but is systematically transformed into decision-relevant inputs.

To improve methodological transparency, a detailed mapping between the original dataset variables, questionnaire items, and the five evaluation criteria is provided in Table 1. It identifies the variables used for each criterion, the coding procedure, and the aggregation method applied to construct the criterion scores. This mapping ensures that the transformation from raw data to decision-making criteria is explicit and reproducible.

**Table 1:** Mapping of dataset variables and questionnaire items to evaluation criteria.

Criterion	Source variables/items	Coding	Aggregation
C1 mental health need and expected effectiveness	Depression (dep), anxiety (anx), somatization (som), hostility (hos)	The clinical symptom variables were standardized using z-score normalization and directionally transformed by multiplying the standardized values by minus one so that lower symptom severity corresponded to higher criterion values representing better expected intervention performance	Mean composite score
C2 user engagement	S-series engagement-related items	Higher scores indicate greater engagement	Mean score
C3 accessibility	Accessibility-related questionnaire items	Higher scores indicate easier access to services	Mean score
C4 cost efficiency	Cost and affordability questionnaire items	Higher scores indicate better perceived cost efficiency	Mean score
C5 privacy	Privacy and confidentiality questionnaire items	Higher scores indicate greater perceived privacy protection	Mean score

For symptom-based variables, lower clinical symptom scores indicate better mental health status. Therefore, after normalization, the values were directionally transformed so that all evaluation criteria followed a consistent benefit orientation, where higher values represented more favorable performance within the proposed decision support framework.

Following the dataset interpretation, primary data are collected via a questionnaire survey. A total of 200 university students from different academic groups are invited to participate. The questionnaire is designed to capture perceptions of mental health support systems, as well as factors related to accessibility, engagement, and perceived effectiveness. Zhengzhou Business University in China confirmed that all questionnaires and the online protocol were completed and that the data obtained are confidentially retained to preserve the anonymity of the respondents. Participants were recruited through voluntary convenience sampling. The survey was distributed to 200 university students from various academic groups to capture a broad range of perspectives on mental health support systems. As the study focused on the university student population, respondents were individuals at different stages of higher education and with potentially diverse experiences of academic and mental health support. Because participation was anonymous and the primary objective was the development of a multi-criteria decision-making framework, detailed demographic subgroup analyses were beyond the scope of the present study. Therefore, the findings should be interpreted within the context of the sampled student population.

This study was conducted using an anonymous online questionnaire survey of university students. According to institutional guidelines, this type of voluntary, anonymous survey research did not require formal approval from the ethics committee because no clinical intervention was performed and no personally identifiable information was collected. Before participation, all respondents were provided with information about the study's purpose and the use of the collected data, and informed consent was obtained electronically. Participation was voluntary, and respondents could discontinue the survey at any time. All data were collected and stored in anonymized form and were used solely for research purposes. Because the study involved mental health-related indicators, including depression, anxiety, somatization, and hostility, only aggregated results were reported, and no individual participant could be identified.

The collected responses are integrated into the dataset to strengthen the study's empirical basis. An expert panel is then involved to validate the selection of criteria and structure the decision framework. Experts in mental health and decision-making review the mapped criteria derived from the dataset and questionnaire responses. Their role is to ensure that each criterion is conceptually sound and relevant for evaluating support systems. In addition, expert judgments are used in later stages to construct pairwise comparisons for criteria weighting. The expert panel consisted of five specialists, including two university counseling psychologists, two mental health researchers, and one expert in multi-criteria decision-making. The panel reviewed the proposed criteria, validated their relevance, and participated in the pairwise comparison process used for criteria weighting and alternative evaluation.

After these steps, the processed data are organized into a decision matrix defined as Equation 1


$$\begin{array}{l} X=\left[x_{ij}\right] \end{array}$$ (1)

where *x*_*ij*_ represents the aggregated performance score of the alternative *i* under criterion *j*. To ensure comparability across criteria, normalization is applied as Equation 2


$$\begin{array}{l} r_{ij}=\frac{x_{ij}}{\sqrt{\sum_{i=1}^{m}x_{ij}^{2}}} \end{array}$$ (2)

**Figure 2:**
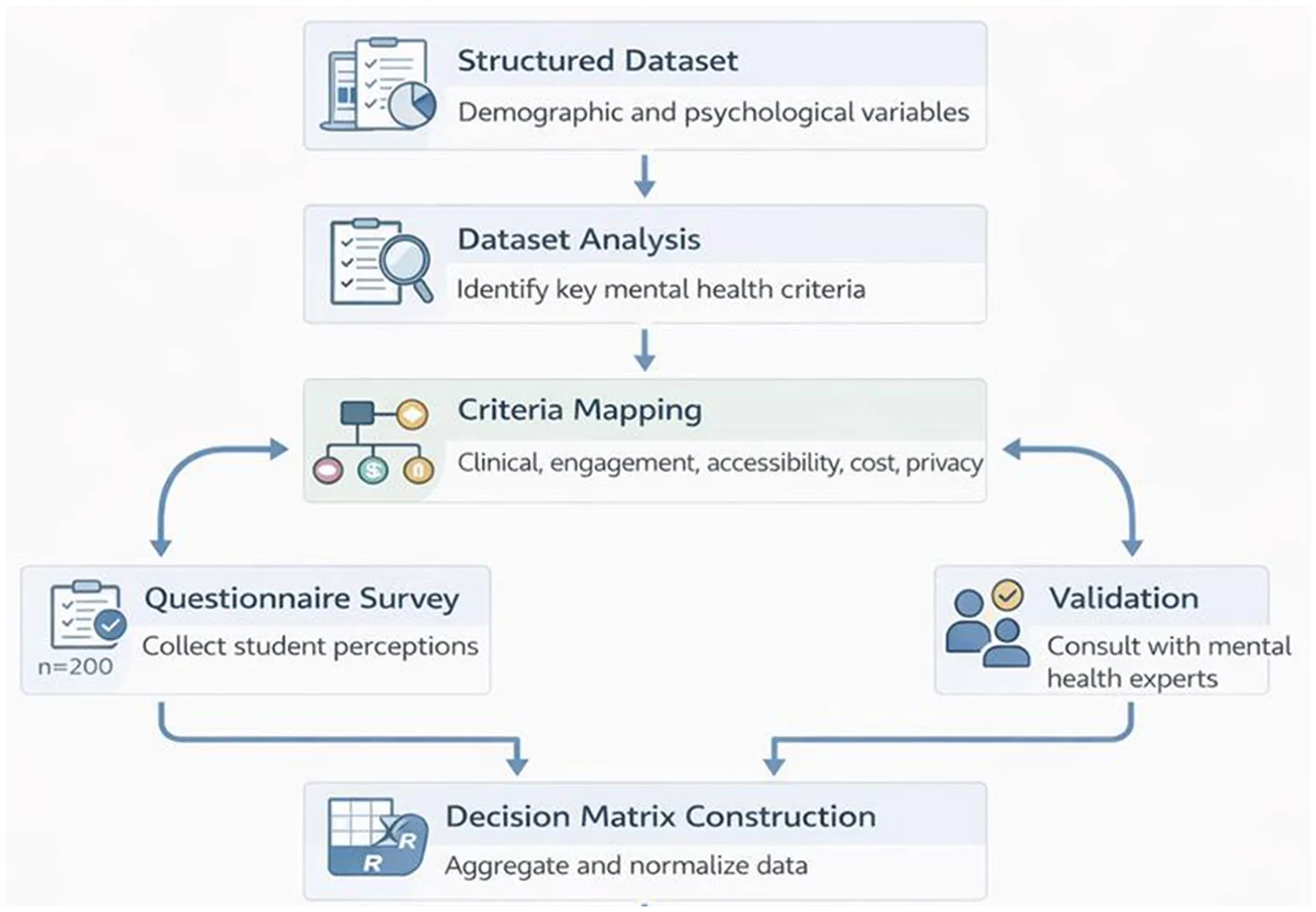
Research design and data collection process illustrating dataset interpretation, criteria mapping, questionnaire-based data collection, expert validation, and construction of the normalized decision matrix for the AHP–TOPSIS analysis.

The overall process, including dataset interpretation, criteria mapping, questionnaire-based data collection, and expert validation, is illustrated in Figure 2. This structured approach ensures that the dataset is meaningfully translated into inputs suitable for the AHP–TOPSIS framework.

#### 2.1.1 Questionnaire reliability assessment

Because several evaluation criteria were derived from multiple questionnaire items, the questionnaire's internal consistency was assessed before aggregating item responses into composite criterion scores. Internal consistency was evaluated using Cronbach's alpha (α), which is widely employed to assess the extent to which items assigned to the same construct measure a common underlying concept.

Cronbach's alpha was calculated separately for each multi-item construct corresponding to the five evaluation criteria. An alpha coefficient of 0.70 or higher was considered indicative of acceptable reliability for exploratory research, while values above 0.80 indicated good internal consistency. Item responses were aggregated into mean composite scores only after satisfactory reliability had been confirmed. This procedure ensured that the questionnaire-derived inputs incorporated into the AHP–TOPSIS decision-making framework exhibited adequate measurement consistency before subsequent weighting and ranking analyses. The reliability results are presented in Table 2.

**Table 2:** Internal consistency reliability of the questionnaire constructs.

Criterion	Number of items	Cronbach's α	Interpretation
C1 mental health need and expected effectiveness	4	0.89	Excellent
C2 user engagement	5	0.86	Good
C3 accessibility	4	0.84	Good
C4 cost efficiency	3	0.81	Good
C5 privacy	4	0.88	Good
Overall questionnaire	20	0.87	Good

As shown in Table 2, all questionnaire constructs achieved Cronbach's alpha coefficients greater than the recommended threshold of 0.70, indicating satisfactory internal consistency. These results support the aggregation of individual questionnaire items into composite scores representing the five evaluation criteria. The questionnaire therefore provides a reliable empirical basis for constructing the decision criteria subsequently used in the AHP weighting process and TOPSIS ranking.

### 2.2 Criteria selection and weighting using AHP

The identification and weighting of evaluation criteria are performed using the Analytic Hierarchy Process. The criteria are derived directly from the dataset through systematic interpretation of variables and are further supported by questionnaire responses. The dataset includes multiple psychological and behavioral indicators, such as depression (dep*), anxiety (anx*), somatization (som*), hostility (hos*), and structured response items (S-series). These variables are grouped into higher-level constructs to form decision criteria. The final set of criteria is reviewed and approved by the expert panel to ensure conceptual validity and relevance. The criteria are defined as follows:

- *C1: Mental Health Need and Expected Effectiveness:* this criterion is derived from psychological symptom variables, including depression, anxiety, somatization, and hostility scores contained in the dataset. These indicators are used to characterize the level of mental health need among students rather than to directly measure intervention outcomes. Within the proposed decision-making framework, higher levels of identified need are combined with expert and literature-based assessments of each support alternative's expected ability to address those needs. Consequently, this criterion should be interpreted as an assessment of mental health need and anticipated intervention effectiveness rather than observed clinical effectiveness.
- *C2: User Engagement:* this criterion is constructed from behavioral and response-based variables, particularly the S-series items in the dataset. These variables reflect the level of student participation, responsiveness, and interaction with support mechanisms. Higher engagement indicates that students are more actively using mental health services, which is essential for achieving positive outcomes. The expert panel validates that engagement is a critical factor influencing the success of both digital and traditional interventions.
- *C3: Accessibility:* this criterion is inferred from variables that indicate how easily students can access mental health support services. It includes aspects such as frequency of use, availability, and perceived ease of reaching support. These elements are derived from responses to selected datasets and questionnaire inputs. Accessibility refers to whether services are accessible without significant barriers. The expert panel approves this criterion as essential for evaluating the practicality and inclusiveness of support systems.
- *C4: Cost Efficiency:* this criterion is derived from questionnaire-based perceptions related to affordability and resource utilization. Although not directly represented by a single dataset variable, it is constructed by integrating relevant survey responses with contextual interpretation. It reflects the balance between the benefits of mental health services and their associated costs. The expert panel confirms that cost efficiency is an important consideration, particularly in university settings where resource constraints may influence service adoption.
- *C5: Privacy:* this criterion is supported by questionnaire items that capture students' perceptions of confidentiality, data protection, and trust in mental health services. It reflects the extent to which students feel secure when sharing sensitive information. Privacy is particularly important in digital platforms where concerns about data security may affect usage. The expert panel validates this criterion as a key factor influencing user acceptance and continued engagement.

**Figure 3:**
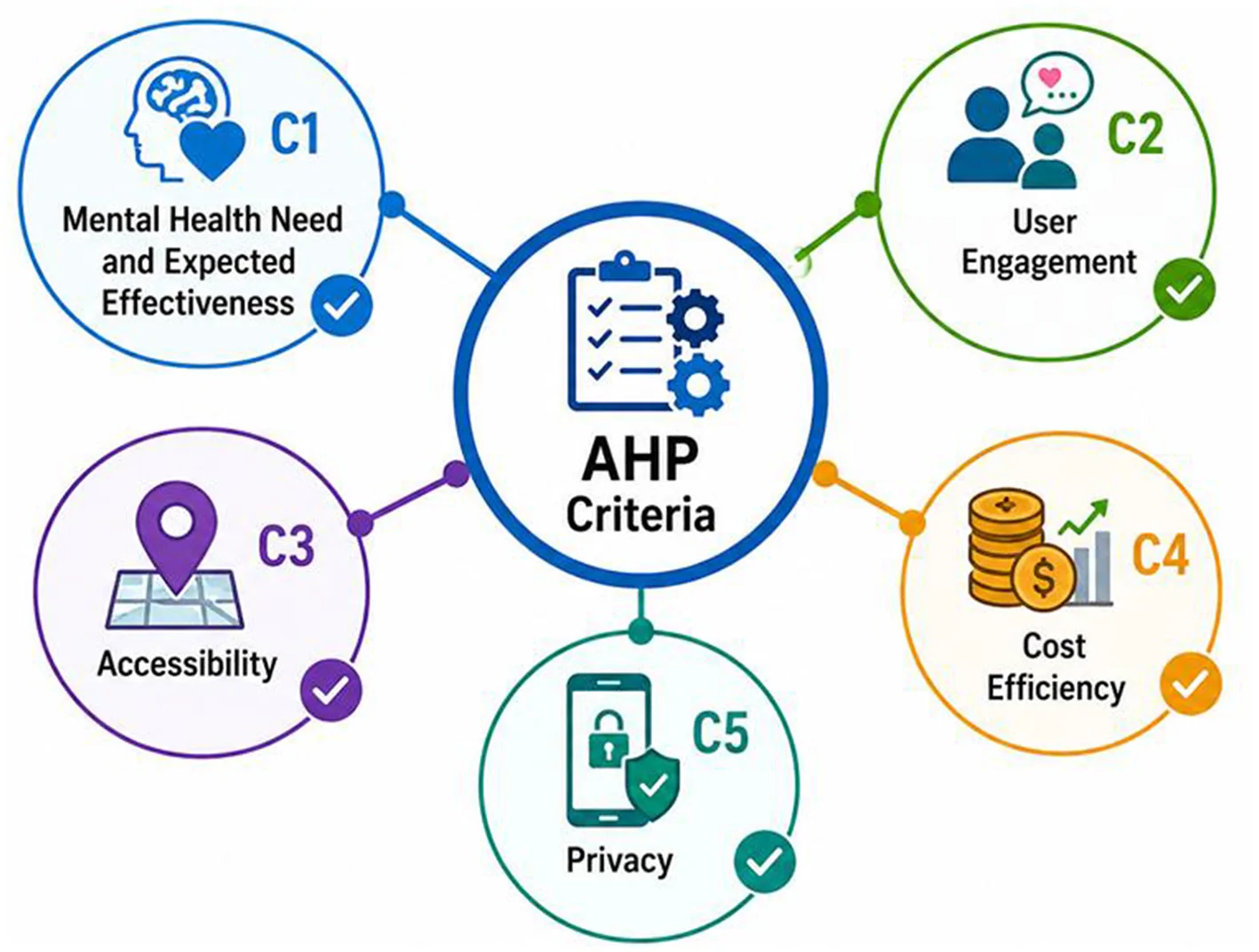
Hierarchical representation of AHP evaluation criteria for mental health support systems, illustrating five key constructs: Mental Health Need and Expected Effectiveness (C1), user engagement (C2), accessibility (C3), cost efficiency (C4), and privacy (C5).

These five criteria represent aggregated constructs derived from the dataset and refined through expert validation. These aggregated constructs were developed to support multi-criteria decision making rather than to establish new psychometric latent variables. Consequently, formal measurement validation procedures, including exploratory factor analysis, confirmatory factor analysis, and reliability assessment, were beyond the scope of the present study and are recommended for future research. Their hierarchical relationship with the overall objective is illustrated in Figure 3.

Following the definition of the criteria, the expert panel performs pairwise comparisons to determine the relative importance of each criterion. The pairwise comparison matrix was developed based on evaluations provided by the expert panel. Individual pairwise comparison judgments were collected using Saaty's nine-point scale. To obtain a single-group comparison matrix, the individual judgments were aggregated using the geometric mean. The aggregated pairwise comparison matrix was normalized by dividing each element in a column by the sum of that column. The priority weight for each criterion was then calculated as the arithmetic mean of the normalized values across each row. The complete normalized matrix, column sums, row-average calculations, and resulting priority vector are provided in the Supplementary material to enable independent verification. Consistency was evaluated using the maximum eigenvalue (λ max), the Consistency Index (CI), and the Consistency Ratio (CR). A CR value below 0.10 was considered acceptable. Consistency was subsequently evaluated using the consistency index and consistency ratio to ensure the reliability of expert judgments.

The comparison matrix is defined as Equation 3


$$\begin{array}{l} A=\left[a_{ij}\right] \end{array}$$ (3)

where *a*_*ij*_ represents the relative importance of the criterion *i* compared to the criterion *j*. The matrix is normalized as Equation 4


$$\begin{array}{l} {ã}_{i}=\frac{a_{ij}}{\sum_{i=1}^{n}a_{ij}} \end{array}$$ (4)

and the criteria weights are calculated as Equation 5


$$\begin{array}{l} w_{i}=\frac{1}{n}\overset{n}{\underset{j=1}{\sum }}{ã}_{ij} \end{array}$$ (5)

To ensure consistency, the consistency index and ratio are computed as Equations 6 and 7


$$\begin{array}{l} CI=\frac{\lambda_{\max}-n}{n-1} \end{array}$$ (6)

$$\begin{array}{l} CR=\frac{CI}{RI} \end{array}$$ (7)

A consistency ratio within the acceptable threshold indicates reliable expert judgments. The resulting weights are then used in the subsequent TOPSIS analysis.

### 2.3 AHP–TOPSIS model implementation and ranking

After determining the criteria weights using the Analytic Hierarchy Process, the alternatives are ranked using TOPSIS. This stage integrates the processed dataset, criteria mapping, and expert-derived weights into a unified evaluation framework.

The alternatives evaluated in this study were not directly represented as categories within the dataset. Rather than assigning individual respondents to specific intervention groups, the alternatives were defined as decision options based on evidence from the literature and validated through expert consultation. Teletherapy (A1) was selected because it represents digital mental health support delivered through online platforms and remote communication technologies. In-person counseling (A2) was included as the conventional model of mental health service delivery based on direct face-to-face interaction between students and mental health professionals. Blended care (A3) was incorporated as an integrated approach that combines digital interventions with traditional counseling services. These three alternatives were chosen because they represent the most common service delivery models currently discussed and implemented in higher education mental health support. The dataset and questionnaire responses were used to identify student needs and establish the evaluation criteria, whereas the performance of the alternatives was assessed against these criteria through expert evaluation informed by empirical findings and existing evidence from the literature.

For each alternative, relevant variables from the dataset are aggregated under each criterion (C1–C5), and mean scores are computed. These aggregated values form the decision matrix, as previously defined in Equation 1, and normalization is performed using Equation 2.

The weighted normalized decision matrix is then constructed as Equation 8


$$\begin{array}{l} v_{ij}=w_{j}\cdot r_{ij} \end{array}$$ (8)

where *w*_*j*_ represents the weight of the criterion *j* obtained from the AHP process. The positive ideal solution and negative ideal solution are determined as Equations 9 and 10


$$\begin{array}{l} A^{+}=\left(\max\left(v_{ij}\right)\right) \end{array}$$ (9)

$$\begin{array}{l} A^{-}=\left(\min\left(v_{ij}\right)\right) \end{array}$$ (10)

The separation distances from the ideal and negative ideal solutions are calculated as Equations 11 and 12


$$\begin{array}{l} S_{i}^{+}=\sqrt{\overset{n}{\underset{j=1}{\sum }}{\left(v_{ij}-A_{j}^{+}\right)}^{2}} \end{array}$$ (11)

$$\begin{array}{l} S_{i}^{-}=\sqrt{\overset{n}{\underset{j=1}{\sum }}{\left(v_{ij}-A_{j}^{-}\right)}^{2}} \end{array}$$ (12)

Finally, the relative closeness to the ideal solution is computed as Equation 13


$$\begin{array}{l} C_{i}=\frac{S_{i}^{-}}{S_{i}^{+}+S_{i}^{-}} \end{array}$$ (13)

The alternatives are ranked based on the values of *C*_*i*_, where higher values indicate better performance. This process enables a comparative evaluation of A1, A2, and A3 by integrating dataset-driven scores and expert-based weights.

### 2.4 Validation and consistency analysis

The reliability of the proposed AHP–TOPSIS framework is evaluated through consistency verification and explicit sensitivity analysis. This step ensures that the derived results are both logically consistent and robust under varying conditions. The consistency of the criteria weights obtained using the Analytic Hierarchy Process is first examined. The pairwise comparison matrix is assessed using the consistency index and consistency ratio, as defined in Equations 6 and 7. A consistency ratio below the acceptable threshold confirms that expert judgments are coherent and suitable for further analysis. To further validate the model's robustness, a sensitivity analysis is conducted by systematically varying the criterion weights. Let the original weight of a criterion be *w*_*j*_. A variation is introduced as Equation 14


$$\begin{array}{l} w_{j}^{\prime }=w_{j}+\Delta w_{j} \end{array}$$ (14)

where Δ*w*_*j*_ represents a small change applied to the weight of the criterion *j*. To maintain normalization, the adjusted weights are recalculated as Equation 15


$$\begin{array}{l} \overset{n}{\underset{j=1}{\sum }}w_{j}^{\prime }=1 \end{array}$$ (15)

Using these modified weights, the weighted normalized matrix is recomputed [as defined in Equation 8], and the relative closeness values are recalculated as Equation 16


$$\begin{array}{l} C_{i}\prime =\frac{S_{i}^{-\prime }}{S_{i}^{+\prime }+S_{i}^{-\prime }} \end{array}$$ (16)

where $C_{i}\prime$ represents the updated closeness coefficient for alternative *i*, and $S_{i}^{{+}^{\prime }}$, $S_{i}^{{-}^{\prime }}$ denote the updated separation distances under the new weight set. The changes in ranking are then evaluated by comparing $C_{i}\prime$ with the original *C*_*i*_. If the ranking order of alternatives (A1–A3) remains unchanged across multiple weight variations, the model is considered stable. To further examine model robustness, additional stress-test scenarios were considered. These scenarios included substantial increases and decreases in the weights of the most influential criteria, alternative weighting distributions that reduced the dominance of the highest-ranked criterion, and moderate perturbations in alternative performance scores. The objective was to evaluate whether the ranking of alternatives remained stable under less favorable assumptions and increased uncertainty.

Significant changes in ranking indicate sensitivity to specific criteria, which are then further examined. In addition, scenario-based sensitivity analysis is performed by increasing and decreasing the weight of each criterion (C1–C5) individually while proportionally adjusting the remaining weights. This allows identification of the most influential criteria affecting the final decision. Finally, the expert panel reviews the sensitivity analysis results to confirm that the model's observed behavior aligns with practical expectations. This combined approach ensures that the proposed framework is not only consistent but also robust under varying decision conditions.

## 3 Results and discussions

### 3.1 Criteria weights and consistency results

The criteria weights are derived using the Analytic Hierarchy Process by integrating questionnaire responses with expert panel judgments. The questionnaire completed by 200 students provides the empirical basis for understanding the relative importance of different aspects of mental health support systems. Each criterion (C1–C5) is represented by a set of questionnaire items and dataset variables, which are first aggregated to obtain mean scores reflecting students' perceptions.

These aggregated scores are then interpreted to guide the construction of the pairwise comparison matrix. Specifically, criteria with higher average scores and stronger representation in the dataset are considered more important during expert evaluation. The expert panel uses this data-driven insight to assign relative importance values using the standard AHP scale. For example, the dataset's higher reported importance of psychological improvement leads to stronger preference values for C1 over other criteria. This process ensures that the pairwise comparisons are not arbitrary but are grounded in both dataset patterns and questionnaire responses. The resulting pairwise comparison matrix is presented in Table 3.

**Table 3:** Pairwise comparison matrix for evaluating the relative importance of mental health support criteria.

Criteria	C1	C2	C3	C4	C5
C1	1	3	4	5	6
C2	1/3	1	2	3	4
C3	1/4	1/2	1	2	3
C4	1/5	1/3	1/2	1	2
C5	1/6	1/4	1/3	1/2	1

Table 3 presents the aggregated pairwise comparison matrix obtained after combining the judgments of the five experts using the geometric mean method. The matrix served as the basis for normalization, weight estimation, and consistency evaluation. The dominance of C1 reflects the importance assigned to addressing identified mental health needs within the student population and the expected capacity of support systems to respond to those needs. The matrix is normalized column-wise to remove scale effects. The normalized values are shown in Table 3. The criteria weights were obtained by averaging the normalized values across each row of the normalized pairwise comparison matrix. The complete calculation procedure, including the normalized matrix, row averages, and consistency verification, is reported in Supplementary Tables S3–S5.

The normalization process reflects proportional importance across criteria. Higher C1 values across columns indicate that both the dataset interpretation and the questionnaire responses consistently emphasize clinical outcomes. The criteria weights are then obtained by averaging the normalized values across each row. The results are presented in Table 4.

**Table 4:** Criteria weights representing the relative importance of mental health support evaluation factors.

Criteria	Weight (w_i_)
C1	0.4847
C2	0.2268
C3	0.1431
C4	0.0888
C5	0.0566

Table 4 shows that C1 has the highest weight, followed by C2 and C3. This indicates that improvements in mental health and user interaction are the most influential factors, as supported by both dataset trends and questionnaire responses. Lower weights for C4 and C5 suggest that cost and privacy, while relevant, are secondary considerations in this context. To ensure that the derived weights are reliable, consistency analysis is performed. The results are summarized in Table 5.

**Table 5:** Consistency results validating the reliability of the AHP pairwise comparisons.

Parameter	Value
λmax	5.0990
Consistency index (CI)	0.0248
Random index (RI)	1.1200
Consistency ratio (CR)	0.0221
Interpretation	Acceptable consistency (CR < 0.10)

The consistency ratio is below the acceptable threshold, indicating that the pairwise comparisons are logically consistent. This confirms that integrating dataset interpretation, questionnaire aggregation, and expert judgment yields stable, reliable criteria weights. Overall, this step demonstrates how raw questionnaire data and dataset variables are systematically interpreted, aggregated, and transformed into weighted decision criteria, forming a critical foundation for the subsequent ranking analysis.

### 3.2 Ranking of alternatives using TOPSIS

The ranking of alternatives is performed using TOPSIS, integrating the criteria weights obtained in Section 3.1 with the dataset-derived performance values. The alternatives are defined as A1 (Teletherapy), A2 (In-person Counseling), and A3 (Blended Care Model).

The performance scores used to construct the decision matrix were obtained through expert evaluation rather than direct observation of intervention outcomes. The five-member expert panel independently assessed teletherapy (A1), in-person counseling (A2), and blended care (A3) against the five evaluation criteria after reviewing the questionnaire findings, interpretation of the SCL-90 dataset, and relevant literature on university mental health support systems. Individual evaluations were subsequently aggregated to obtain the final criterion scores used in the TOPSIS analysis. Detailed expert evaluation scores are reported in Supplementary Table S5.

The assigned values reflect the experts' assessment of the relative performance of each alternative under the selected criteria. For example, blended care received higher scores for Mental Health Need and Expected Effectiveness, User Engagement, and Accessibility because the integration of digital and face-to-face support was considered better suited to addressing diverse student needs while maintaining service accessibility and engagement. Teletherapy received the highest score for Cost Efficiency due to its scalability and lower resource requirements, whereas in-person counseling received relatively strong scores for Mental Health Need and Expected Effectiveness due to the perceived benefits of direct therapeutic interaction. These expert-informed assessments formed the basis of the decision matrix presented in Table 6.

**Table 6:** Aggregated decision matrix representing the performance of alternatives across criteria.

Alternatives	C1	C2	C3	C4	C5
A1	3.8	3.5	4.0	4.2	3.6
A2	4.2	3.2	3.5	3.8	3.4
A3	4.5	4.0	4.3	4.1	4.0

The dataset and questionnaire responses were used to establish the relative importance of the evaluation criteria and to provide empirical context regarding student mental health needs and service preferences. Performance scores for the three alternatives were subsequently determined through expert assessment based on the defined criteria, supported by evidence from the dataset and the literature on university mental health support systems. The constructed decision matrix is presented in Table 5. The values reported in Table 5 were obtained by aggregating criterion-specific assessments for each alternative. For each criterion, performance scores were derived from expert evaluations informed by questionnaire findings, dataset interpretation, and evidence from the literature. Mean values were calculated for each alternative and subsequently normalized prior to applying TOPSIS.

Table 6 presents the expert-assigned performance scores for each alternative under the five evaluation criteria. These values were obtained by aggregating the evaluations of the five-member expert panel, taking into account the questionnaire findings, the interpretation of the SCL-90 dataset, and evidence from the literature. The decision matrix itself was not affected by the recalculation of the AHP weights because it represents the performance of the alternatives rather than the relative importance of the criteria. To eliminate the effect of scale differences among criteria, the decision matrix is normalized using Equation 2. The normalized decision matrix is shown in Table 7.

**Table 7:** Normalized decision matrix showing relative performance of alternatives.

Alternatives	C1	C2	C3	C4	C5
A1	0.5424	0.5449	0.5744	0.6032	0.5771
A2	0.5995	0.4982	0.5026	0.5457	0.5451
A3	0.6423	0.6228	0.6175	0.5888	0.6412

Table 7 indicates that A3 retains the highest normalized values across most criteria, confirming its relative dominance. A2 shows competitive performance in C1 but remains lower in engagement-related aspects. A1 maintains balanced but non-dominant values across all criteria. The normalized values are then weighted using the criteria weights obtained in Table 8. The resulting weighted normalized matrix is presented in Table 9.

**Table 8:** Normalized pairwise comparison matrix representing the relative importance of evaluation criteria.

Criteria	C1	C2	C3	C4	C5
C1	0.5128	0.5902	0.5106	0.4348	0.3750
C2	0.1709	0.1967	0.2553	0.2609	0.2500
C3	0.1282	0.0984	0.1277	0.1739	0.1875
C4	0.1026	0.0656	0.0638	0.0870	0.1250
C5	0.0855	0.0492	0.0426	0.0435	0.0625

**Table 9:** Weighted normalized decision matrix reflecting the relative importance of evaluation criteria.

Alternatives	C1	C2	C3	C4	C5
A1	0.2629	0.1236	0.0822	0.0536	0.0327
A2	0.2906	0.1130	0.0719	0.0485	0.0308
A3	0.3113	0.1413	0.0884	0.0523	0.0363

Table 9 highlights the influence of criteria weights on the evaluation. The dominance of C1 and C2 results in higher weighted values for A3, as it performs strongly in these criteria. A2 benefits from its relatively high Mental Health Need and Expected Effectiveness, but remains limited by lower engagement and accessibility scores. A1 shows comparatively lower weighted contributions, particularly in high-priority criteria. The positive and negative ideal solutions are identified based on the best and worst weighted values, and the separation distances are computed accordingly. The results are presented in Table 10.

**Table 10:** Separation distances used to evaluate alternatives based on ideal and negative ideal solutions.

Alternatives	Distance from ideal (S^+^)	Distance from negative ideal (S^−^)
A1	0.0563	0.0178
A2	0.0372	0.050
A3	0.0013	0.0592

Table 10 shows that A3 has the smallest distance to the positive ideal solution and the largest distance to the negative ideal solution, indicating superior overall performance. In contrast, A1 exhibits the largest distance from the ideal solution, reflecting weaker relative performance. Finally, the relative closeness coefficients are calculated to determine the final ranking. The results are presented in Table 11.

**Table 11:** Relative closeness coefficients and final ranking of mental health support alternatives.

Alternatives	Closeness coefficient (C_i_)	Rank
A1	0.240	3
A2	0.402	2
A3	0.979	1

Table 11 indicates that the blended care model (A3) achieved the highest relative closeness coefficient (0.979), indicating the closest proximity to the positive ideal solution and the greatest distance from the negative ideal solution within the proposed AHP–TOPSIS framework. In-person counseling (A2) ranked second with a closeness coefficient of 0.402, whereas teletherapy (A1) ranked third with a coefficient of 0.240. The higher ranking of the blended care model reflects its comparative performance under the expert-assessed evaluation criteria and weighting structure adopted in the proposed framework and should not be interpreted as evidence of inherent or universal superiority across all clinical or institutional contexts.

### 3.3 Sensitivity analysis and discussion of findings

The robustness of the obtained ranking results is further examined through sensitivity analysis. This analysis evaluates how variations in criteria weights influence the final ranking of alternatives derived using the TOPSIS. Since the weights are initially obtained through the Analytic Hierarchy Process, it is essential to assess whether small or moderate changes in these weights affect the stability of the decision outcomes. The sensitivity analysis is conducted by systematically varying the weights of each criterion (C1–C5) while maintaining the normalization condition. Specifically, each criterion weight is adjusted within a defined range (± 10% or ± 20%), and the remaining weights are adjusted proportionally. For each variation scenario, the TOPSIS procedure is repeated, and new closeness coefficients are computed. The results of weight variation scenarios are summarized in Table 12.

**Table 12:** Sensitivity analysis under criteria weight variations.

Scenario	C1 weight	C2 weight	c3 weight	C4 weight	C5 weight	A1 (CC_i_)	A2 (CC_i_)	A3 (CC_i_)	Ranking order
Base case	0.4847	0.2268	0.1431	0.0888	0.0566	0.240	0.402	0.979	A3 > A2 > A1
S1 (+10% C1)	0.5332	0.2055	0.1295	0.0803	0.0515	0.224	0.389	0.983	A3 > A2 > A1
S2 (−10% C1)	0.4362	0.2482	0.1567	0.0973	0.0616	0.257	0.415	0.974	A3 > A2 > A1
S3 (+20% C2)	0.4566	0.2722	0.1348	0.0833	0.0531	0.248	0.389	0.978	A3 > A2 > A1
S4 (−20% C2)	0.5128	0.1814	0.1515	0.0943	0.0600	0.233	0.41	0.41	A3 > A2 > A1

Table 12 demonstrates that moderate variations in the criteria weights produced only small changes in the TOPSIS closeness coefficients. In all tested scenarios, the ranking order remained unchanged (A3 > A2 > A1). Increasing the weight of the most influential criterion (C1) slightly strengthened the dominance of the blended care model (A3), whereas decreasing the weight of C1 produced a modest improvement in the relative closeness of A1 and A2. Similar behavior was observed when the weight of User Engagement (C2) was varied. These results indicate that the proposed AHP–TOPSIS framework is robust to moderate changes in the weighting structure.

To further examine the influence of individual criteria, a one-at-a-time sensitivity approach is applied, in which each criterion weight is varied independently while the others are proportionally adjusted. The results are presented in Table 13.

**Table 13:** Sensitivity analysis of individual criteria and their impact on alternative ranking.

Criterion varied	Range tested	Ranking stability	Most affected alternative
C1	± 20%	Stable	A2
C2	± 20%	Stable	A1
C3	± 20%	Stable	A2
C4	± 20%	Stable	A1
C5	± 20%	Stable	A1

Table 13 shows that varying each criterion independently within a ± 20% range did not alter the ranking order of the three alternatives. The largest numerical changes in the closeness coefficients were observed when the weights of C1 (Mental Health Need and Expected Effectiveness) and C2 (User Engagement) were modified, reflecting their relatively higher priority weights in the AHP analysis. However, these variations were insufficient to change the ranking, with blended care (A3) consistently remaining the highest-ranked alternative, followed by in-person counseling (A2) and teletherapy (A1). These findings indicate that the proposed AHP–TOPSIS framework is robust to moderate variations in the relative importance assigned to individual evaluation criteria.

From an interpretative perspective, the results highlight that criteria such as Mental Health Need, Expected Effectiveness, and user engagement play a critical role in shaping the final ranking. Alternatives that perform well in these dimensions tend to maintain higher rankings even under varying conditions. The relatively lower impact of cost efficiency and privacy suggests that, within this dataset and respondent group, outcome-related factors are prioritized over auxiliary considerations. Overall, the sensitivity analysis confirms that the proposed AHP–TOPSIS framework is robust, reliable, and capable of producing stable rankings. The consistency of results across multiple scenarios strengthens the validity of the conclusion that blended care models are the most effective mental health support systems among the evaluated alternatives.

Additional robustness checks were conducted using alternative weighting configurations and moderate variations in criterion performance scores. Although small changes in closeness coefficients were observed, the ranking order remained unchanged, with the blended care model consistently achieving the highest score. These findings suggest that the superiority of the blended care alternative is not solely attributable to the original weighting scheme but is also supported by balanced performance across multiple evaluation criteria.

It should be noted that the sensitivity analysis performed in this study evaluates the stability of the ranking results under selected variations in criteria weights and alternative performance assumptions. The analysis demonstrates that the ranking of alternatives remains stable within the examined scenarios. However, ranking stability should not be interpreted as evidence of the empirical accuracy of the expert-assigned performance scores. Because the decision matrix was derived from expert evaluation informed by questionnaire findings, dataset interpretation, and evidence from the literature, uncertainty associated with expert judgment remains. More advanced robustness analyses, including probabilistic sensitivity analysis, perturbation-based uncertainty modeling, or fuzzy AHP–TOPSIS approaches, may provide a more comprehensive assessment of uncertainty and should be considered in future research.

## 4 Discussions and limitations

The findings of this study provide important insights into the evaluation of mental health support systems among university students using an integrated AHP–TOPSIS framework. The results indicate that the blended care model (A3) consistently ranks highest within the proposed decision-support framework. This outcome suggests that combining digital and traditional approaches offers a more balanced solution, particularly in addressing both Mental Health Need and Expected Effectiveness, as well as user engagement.

The higher ranking of A3 within the framework can be attributed to its ability to integrate the accessibility advantages of digital platforms with the therapeutic depth of face-to-face interaction. Although the blended care model achieved the highest ranking within the proposed decision-support framework, its practical implementation may pose substantial organizational and financial challenges. Successful adoption requires coordinated investment in both digital mental health infrastructure and conventional counseling services, together with appropriate technological support, professional training, system integration, and long-term maintenance. Universities with limited financial or human resources may therefore encounter barriers that are not explicitly represented within the present AHP–TOPSIS framework. Consequently, the preferred alternative identified by the framework should be interpreted in conjunction with institutional capacity, available resources, and implementation feasibility.

The findings may also be interpreted from several theoretical perspectives. From the perspective of user engagement theory, sustained participation is widely recognized as a critical determinant of intervention success, particularly in digital mental health environments where continued interaction influences outcomes. The relatively high importance assigned to user engagement in the present study supports the view that effective mental health services should promote active and continuous student involvement. In addition, the strong performance of the blended care model is consistent with therapeutic alliance theory, which emphasizes the value of meaningful human interaction, trust, and supportive relationships in mental health interventions. By combining digital accessibility with opportunities for direct professional support, blended care may better address both engagement and relationship-based aspects of service delivery. The findings may also be interpreted through digital mental health adoption frameworks, which suggest that acceptance and sustained use of mental health technologies depend on perceived usefulness, accessibility, trust, and user experience. These theoretical perspectives help explain why a balanced approach that integrates digital and traditional support mechanisms achieved the highest ranking within the proposed evaluation framework.

These findings are consistent with recent literature. Studies have shown that multi-criteria decision-making approaches, such as AHP–TOPSIS, provide a reliable and structured evaluation of complex mental health systems, enabling better prioritization of interventions (21). Similarly, recent research applying TOPSIS in mental health intervention selection highlights its effectiveness in identifying optimal programs under multiple criteria and uncertainty (21, 22). Furthermore, contemporary studies on digital mental health interventions emphasize that while digital tools improve accessibility and scalability, user engagement and clinical outcomes are significantly enhanced when combined with traditional support methods (23).

From a theoretical perspective, the study confirms the applicability of multi-criteria decision-making methods in educational and psychological contexts. Recent reviews indicate that AHP has been widely validated for prioritizing psychological and educational factors, reinforcing its suitability for this type of analysis (24). The integration of AHP with TOPSIS reduces subjectivity and improves decision accuracy, a finding also confirmed in other application domains. In this study, the combination of dataset-driven criteria and expert validation enhances the framework's robustness.

Despite these contributions, several limitations are identified. First, the dataset is limited to a specific group of university students, which may restrict the generalizability of the findings. Mental health conditions and service preferences may vary across regions, cultures, and institutional settings. Second, mapping dataset variables to criteria involves some interpretation, which introduces potential subjectivity, even with expert validation.

Another limitation concerns the absence of formal psychometric validation of the proposed evaluation criteria. The criteria were developed through interpretation of the public dataset, questionnaire evidence, and expert validation to support the proposed multi-criteria decision-making framework rather than to establish a new measurement instrument. Another limitation concerns the absence of formal psychometric validation beyond internal consistency assessment. Although questionnaire reliability was examined using Cronbach's alpha, exploratory factor analysis, confirmatory factor analysis, and broader construct validity assessments were beyond the scope of the present study and should be considered in future research.

Third, the sample size of 200 respondents, while adequate for analysis, may not fully capture the diversity of student experiences. Additional limitations should also be acknowledged. The questionnaire data were based on self-reported responses and may therefore be affected by response bias, including social desirability bias and variations in individual perceptions. Furthermore, the study was conducted within a specific higher education context, and cultural, institutional, and regional factors may influence both mental health needs and preferences for support services. Although expert validation was incorporated throughout the AHP process, the weighting procedure remains partially dependent on expert judgment and may therefore be subject to expert-related bias. In addition, the study adopted a cross-sectional design and captured perceptions at a single point in time. Consequently, changes in student mental health conditions, service preferences, and support needs over time could not be examined.

Another limitation arises from the static nature of the classical AHP–TOPSIS methodology. The framework provides a cross-sectional representation of the decision problem by assuming fixed criteria weights and alternative performance throughout the evaluation process. Consequently, temporal changes in student mental health needs, institutional resource availability, and the relative performance of intervention alternatives cannot be explicitly represented. Although this assumption is common in conventional AHP–TOPSIS applications, it may limit the framework's applicability to rapidly evolving decision environments. Future research should investigate dynamic and longitudinal multi-criteria decision-making approaches, including time-dependent weighting schemes, adaptive decision models, and dynamic or fuzzy AHP–TOPSIS extensions, to better capture changes in mental health service needs and resource allocation over time.

An additional limitation is that the evaluated alternatives were defined from the literature and expert consultation rather than being directly observed intervention groups within the dataset. Consequently, the results should be interpreted as a structured decision-support evaluation rather than a direct comparative effectiveness study. Another limitation arises from the deterministic nature of the classical AHP–TOPSIS methodology. Expert judgments are represented by precise numerical values that do not explicitly account for uncertainty, ambiguity, or hesitation that may arise when evaluating complex decision problems, such as university mental health support systems. Although the consistency analysis confirmed that the expert judgments were logically coherent, the adopted framework does not model uncertainty in the decision-making process. Future research should investigate uncertainty-aware multi-criteria decision-making approaches, including fuzzy AHP–TOPSIS, probabilistic sensitivity analysis, interval-valued methods, and stochastic decision models, to provide a more comprehensive representation of expert knowledge and decision uncertainty.

Future research can address these limitations by incorporating larger, more diverse datasets, applying dynamic or longitudinal analysis methods, and integrating advanced decision-making techniques, such as fuzzy or probabilistic models. Moreover, deeper exploration of digital mental health tools, including AI-based systems and personalized interventions, can further enhance the evaluation framework. Overall, the study demonstrates that a data-driven and expert-validated AHP–TOPSIS approach provides a systematic and reliable method for evaluating mental health support systems. The findings support growing evidence that blended care models offer a comprehensive and effective approach to improving student mental health outcomes in higher education settings. In addition, the present study primarily relied on multi-criteria decision-making procedures and descriptive aggregation techniques rather than inferential statistical analyses. While this approach is appropriate for the objectives of the AHP–TOPSIS framework, future research may incorporate complementary inferential methods, such as regression analysis, structural equation modeling, or hypothesis-testing approaches, to further strengthen the empirical validation of mental health support system evaluations.

## 5 Conclusions and future recommendations

This study presented a structured evaluation of mental health support systems for university students using an integrated AHP–TOPSIS decision support framework. The recalculated results indicate that the blended care model (A3) achieved the highest ranking, with a closeness coefficient of 0.979, followed by in-person counseling (A2) with 0.402 and teletherapy (A1) with 0.240. These results indicate that the blended care model provides the most balanced performance across the selected evaluation criteria within the proposed decision-making framework. Although the numerical values were updated following recalculation of the AHP weights and TOPSIS procedure, the overall ranking of the alternatives remained unchanged, which confirms the robustness of the proposed framework.

The AHP analysis identified Mental Health Need and Expected Effectiveness (C1) as the most influential criterion, with a priority weight of 0.4847, followed by User Engagement (C2, 0.2268), Accessibility (C3, 0.1431), Cost Efficiency (C4, 0.0888), and Privacy (C5, 0.0566). The consistency analysis yielded a Consistency Ratio (CR) of 0.0221, well below the acceptable threshold of 0.10, confirming the reliability of the expert judgments. Furthermore, the sensitivity analysis demonstrated that the ranking of alternatives remained stable under ±20% variations in the criteria weights, providing additional evidence of the robustness of the proposed decision support framework.

Within the proposed AHP–TOPSIS framework, the blended care model consistently emerged as the preferred alternative because it achieved balanced performance across mental health need and expected effectiveness, user engagement, accessibility, cost efficiency, and privacy. The findings should be interpreted as a comparative evaluation based on the specified criteria and weighting structure, rather than as direct evidence of superior clinical effectiveness or causal improvement in student mental health outcomes. Instead, the proposed framework provides a transparent and systematic method to support decision-making in selecting mental health service models in higher education. The comparative assessment of the intervention alternatives was based on structured expert evaluation informed by the empirical data and the literature, rather than direct observation of intervention outcomes.

Future research should validate the proposed framework using larger and more diverse datasets from multiple universities and cultural settings to improve its generalizability. Longitudinal studies are also recommended to examine changes in student mental health needs and service preferences over time. In addition, advanced multi-criteria decision-making approaches, such as fuzzy AHP–TOPSIS, probabilistic AHP, or stochastic decision models, could be incorporated to better represent uncertainty in expert judgments and alternative evaluations. Practical implementation of the framework may support university administrators and policy makers in prioritizing investments in blended mental health services, strengthening digital infrastructure, and integrating technology-based and face-to-face interventions. Nevertheless, the present findings should be interpreted within the scope of the adopted decision support framework and should not be considered direct evidence of intervention effectiveness.

## Data Availability

The original contributions presented in the study are included in the article/Supplementary material, further inquiries can be directed to the corresponding author.

## References

[1] Pinchuk I Feldman I Seleznova V Virchenko V. Braving the dark: mental health challenges and academic performance of Ukrainian university students during the war. Soc Psychiatry Psychiatr Epidemiol. (2025) 60:2505–16. doi: 10.1007/s00127-025-02867-7 40029404 PMC12449376

[2] Wang Y Zhang J Huang L Li X Zhong Y. Association between healthy lifestyle choices and mental health among students: a cross sectional study. BMC Public Health. (2025). 25:247. doi: 10.1186/s12889-025-21482-4 39838333 PMC11749073

[3] Li J Li J Wang C Verbeek FJ Schultz T Liu H. Outlier detection using iterative adaptive mini-minimum spanning tree generation with applications on medical data. Front Physiol. (2025) 14:1233341. doi: 10.3389/fphys.2023.1233341 PMC10613083 37900945

[4] Yang M Li X Qin X Tian X Zhang H Wen H. The relationship between perceived academic stress and college students' employment anxiety: the mediating role of psychological resilience. Front Psychiatry. (2025) 16:1602808. doi: 10.3389/fpsyt.2025.1602808 40530071 PMC12170629

[5] Woodward MJ McGettrick CR Dick OG Ali M Teeters JB. Time spent on social media and associations with mental health in young adults: Examining TikTok, Twitter, Instagram, Facebook, Youtube, Snapchat, and Reddit. J Technol Behav Sci. (2025) 10:661–71. doi: 10.1007/s41347-024-00474-y 41050921 PMC12488834

[6] Li YK Xiao CL Ren H Li WR Guo Z Luo JQ. Unraveling the effectiveness of new media teaching strategies in pharmacology education under different educational backgrounds: Insightsfrom 6447 students. Eur J Pharmacol. (2025) 989:177255. doi: 10.1016/j.ejphar.2025.177255 39788408

[7] Hofmann SG Asnaani A Vonk IJ Sawyer AT Fang A. The efficacy of cognitive behavioral therapy: A review of meta-analyses. Cognit Ther Res. (2012) 36:427–440. doi: 10.1007/s10608-012-9476-1 23459093 PMC3584580

[8] Madrid-Cagigal A Kealy C Potts C Mulvenna MD Byrne M Barry MM . Digital mental health interventions for university students with mental health difficulties: A systematic review and meta-analysis. Early Interv Psychiatry. (2025) 19:e70017. doi: 10.1111/eip.70017 40033658 PMC11876723

[9] Huang C . How does social support detected automatically in discussion forums relate to online learning burnout? The moderating role of students' self-regulated learning. Comput Educ. (2025) 227:105213. doi: 10.1016/j.compedu.2024.105213

[10] Linardon J Cuijpers P Carlbring P Messer M Fuller-Tyszkiewicz M. The efficacy of app-supported smartphone interventions for mental health problems: a meta-analysis of randomized controlled trials. World Psychiatry. (2019) 18:325–36. doi: 10.1002/wps.20673 31496095 PMC6732686

[11] Fleming T Bavin L Lucassen M Stasiak K Hopkins S Merry S. Beyond the trial: systematic review of real-world uptake and engagement with digital self-help interventions for depression, low mood, or anxiety. J Med Internet Res. (2018) 20:e199. doi: 10.2196/jmir.9275 29875089 PMC6010835

[12] Cuijpers P Karyotaki E Reijnders M Ebert DD. Was Eysenck right after all? A reassessment of the effects of psychotherapy for adult depression. Epidemiol Psychiatr Sci. (2019) 28:21–30. doi: 10.1017/S2045796018000057 29486804 PMC6999031

[13] Perski O Blandford A West R Michie S. Conceptualising engagement with digital behaviour change interventions: a systematic review using principles from critical interpretive synthesis. Transl Behav Med. (2017) 7:254–67. doi: 10.1007/s13142-016-0453-1 27966189 PMC5526809

[14] Torous J Bucci S Bell IH Kessing LV Faurholt-Jepsen M Whelan P . The growing field of digital psychiatry: current evidence and the future of apps, social media, chatbots, and virtual reality. World Psychiatry. (2021) 20:318–35. doi: 10.1002/wps.20883 34505369 PMC8429349

[15] Bruffaerts R Mortier P Auerbach RP Alonso J Hermosillo De la Torre AE Cuijpers P . Lifetime and 12-month treatment for mental disorders and suicidal thoughts and behaviors among first year college students. Int J Methods Psychiatr Res. (2019) 28:e1764. doi: 10.1002/mpr.1764 30663193 PMC6877191

[16] Naslund JA Bondre A Torous J Aschbrenner KA. Social media and mental health: benefits, risks, and opportunities for research and practice. J Technol Behav Sci. (2020) 5:245–57. doi: 10.1007/s41347-020-00134-x 33415185 PMC7785056

[17] Wang Q . The burden of travel for care and its influencing factors in China: An inpatient-based study of travel time. J Transp Health. (2022) 25:101353. doi: 10.1016/j.jth.2022.101353

[18] Erbe D Eichert HC Riper H Ebert DD. Blending face-to-face and internet-based interventions for the treatment of mental disorders in adults: systematic review. J Med Internet Res. (2017) 19:e306. doi: 10.2196/jmir.6588 28916506 PMC5622288

[19] Tzeng GH Huang JJ. Multiple Attribute Decision Making: Methods and Applications. Boca Raton, FL: CRC Press (2011). doi: 10.1201/b11032

[20] Chaube S Pant S Kumar A Uniyal S Singh MK Kotecha K . An overview of multi-criteria decision analysis and the applications of AHP and TOPSIS methods. Int J Math Eng Manag Sci. (2024) 9:581. doi: 10.33889/IJMEMS.2024.9.3.030

[21] Amarnath N Chandregowda AY Kodipalli A Rajanna RB Soma B Narayanappa G . Analysis of factors affecting mental illness and social stress in young adults using fuzzy TOPSIS and AHP methods. Melville, NY: AIP Publishing LLC.

[22] Teja MR Uttarkar C Kodipalli A BR R Soma B. Analysis of Mental and Physical Health for Personality Development using Fuzzy AHP and Fuzzy Topsis. New York, NY: IEEE.

[23] Liverpool S Mc Donagh C Feather J Uzondu C Howarth M Bannerman F . Updates on digital mental health interventions for children and young people: systematic overview of reviews. Eur Child Adolesc Psychiatry. (2025) 34:2961–74. doi: 10.1007/s00787-025-02722-9 40278894 PMC12592321

[24] Xu X Liu R Serrano ED. An analytic hierarchy process–based prioritization of psychological factors influencing academic performance among university students in China. Sci Rep. (2026). doi: 10.1038/s41598-026-38343-8 PMC12923886 41639208

