# Supplementary material for: A hybrid AHP–TOPSIS decision-support framework for evaluating mental health support alternatives in higher education
Source: Front Public Health. 2026 Aug 12;14:1861300. doi: 10.3389/fpubh.2026.1861300 (PMC13506445; doi:10.3389/fpubh.2026.1861300)
Supplement: Supplementary file 1 [file Table_1.docx]

This supplementary appendix provides the intermediate calculations and supporting information used in implementing the AHP–TOPSIS framework. The materials are intended to improve transparency and facilitate replication of the analysis procedure.

Table S1 summarizes the composition of the expert panel involved in criteria validation and pairwise comparison assessment. The panel was selected to ensure expertise in mental health services, psychology, and multi-criteria decision-making.

Table S1. Expert Panel Characteristics

| Expert | Expertise Area | Years of Experience | Role in Study |
| --- | --- | --- | --- |
| E1 | University Counseling Psychology | 12 | Criteria validation and pairwise comparison |
| E2 | Clinical Psychology | 10 | Criteria validation and pairwise comparison |
| E3 | Mental Health Research | 9 | Criteria validation and pairwise comparison |
| E4 | Student Wellbeing Services | 8 | Criteria validation and pairwise comparison |
| E5 | Multi-Criteria Decision Analysis | 11 | Criteria validation and pairwise comparison |

Table S2 presents the individual pairwise comparison scores provided by the experts and the corresponding geometric mean values used to construct the final group comparison matrix. The geometric mean aggregation method was adopted because it is widely recommended for group AHP applications.

Table S2. Aggregation of Expert Judgments Using Geometric Mean

| Comparison | E1 | E2 | E3 | E4 | E5 | Geometric Mean |
| --- | --- | --- | --- | --- | --- | --- |
| C1 vs C2 | 3 | 4 | 3 | 2 | 3 | 2.94 |
| C1 vs C3 | 4 | 5 | 4 | 3 | 4 | 3.96 |
| C1 vs C4 | 5 | 5 | 6 | 4 | 5 | 4.95 |
| C1 vs C5 | 6 | 7 | 6 | 5 | 6 | 5.95 |
| C2 vs C3 | 2 | 2 | 3 | 2 | 2 | 2.18 |
| C2 vs C4 | 3 | 4 | 3 | 3 | 3 | 3.18 |
| C2 vs C5 | 4 | 4 | 5 | 4 | 4 | 4.18 |
| C3 vs C4 | 2 | 2 | 2 | 2 | 3 | 2.17 |
| C3 vs C5 | 3 | 3 | 3 | 2 | 3 | 2.77 |
| C4 vs C5 | 2 | 2 | 2 | 2 | 2 | 2.00 |

**Table S3.** Normalized Pairwise Comparison Matrix

| Criteria | C1 | C2 | C3 | C4 | C5 |
| --- | --- | --- | --- | --- | --- |
| C1 | 0.5128 | 0.5902 | 0.5106 | 0.4348 | 0.3750 |
| C2 | 0.1709 | 0.1967 | 0.2553 | 0.2609 | 0.2500 |
| C3 | 0.1282 | 0.0984 | 0.1277 | 0.1739 | 0.1875 |
| C4 | 0.1026 | 0.0656 | 0.0638 | 0.0870 | 0.1250 |
| C5 | 0.0855 | 0.0492 | 0.0426 | 0.0435 | 0.0625 |

The aggregated pairwise comparison matrix was normalized by dividing each element by the sum of its corresponding column. The resulting normalized matrix was used to derive the priority vector by averaging the normalized values across each row.

Table S4 presents the priority vectors and final criteria weights derived from the normalized pairwise comparison matrix. These weights were subsequently used in the TOPSIS evaluation stage.

**Table S4.** Derivation of the Priority Vector and Final Criteria Weights

| Criterion | Row Average (Priority Vector) | Final Weight |
| --- | --- | --- |
| C1. Mental Health Need and Expected Effectiveness | 0.4847 | 0.4847 |
| C2. User Engagement | 0.2268 | 0.2268 |
| C3. Accessibility | 0.1431 | 0.1431 |
| C4. Cost Efficiency | 0.0888 | 0.0888 |
| C5. Privacy | 0.0566 | 0.0566 |

**Total = 1.0000**

The priority vector was obtained by averaging the normalized values across each row of the normalized pairwise comparison matrix (Table S2). These values represent the final AHP criteria weights used in the subsequent TOPSIS analysis. The weights sum to 1.0000, confirming correct normalization of the priority vector. Table S5 reports the consistency assessment of the aggregated pairwise comparison matrix. The calculated consistency ratio remained below the accepted threshold of 0.10, indicating satisfactory consistency of expert judgments.

**Table S5.** Consistency Verification of the AHP Pairwise Comparison Matrix

| Parameter | Value |
| --- | --- |
| Maximum Eigenvalue (λmax) | 5.0990 |
| Consistency Index (CI) | 0.0248 |
| Random Index (RI) | 1.1200 |
| Consistency Ratio (CR) | 0.0221 |
| Interpretation | Acceptable consistency (CR < 0.10) |

The consistency of the aggregated pairwise comparison matrix was evaluated using the maximum eigenvalue (λmax), the Consistency Index (CI), and the Consistency Ratio (CR). The calculated CR of **0.0221** is well below the recommended threshold of **0.10**, indicating that the expert judgments were internally consistent and suitable for deriving the criteria weights. Consequently, the resulting priority vector was deemed sufficiently reliable for subsequent TOPSIS analysis. Table S6 presents the performance scores assigned to each alternative under the five evaluation criteria. The scores were determined through expert assessment informed by questionnaire findings, dataset interpretation, and evidence from the literature.

**Table S6.** Expert Evaluation Scores for Alternatives under the Selected Evaluation Criteria

| Alternative | Mental Health Need and Expected Effectiveness (C1) | User Engagement (C2) | Accessibility (C3) | Cost Efficiency (C4) | Privacy (C5) |
| --- | --- | --- | --- | --- | --- |
| A1 (Teletherapy) | 3.8 | 3.5 | 4.0 | 4.2 | 3.6 |
| A2 (In-Person Counseling) | 4.2 | 3.2 | 3.5 | 3.8 | 3.4 |
| A3 (Blended Care) | 4.5 | 4.0 | 4.3 | 4.1 | 4.0 |

The performance scores were assigned independently by the five-member expert panel after reviewing the questionnaire findings, interpretation of the SCL-90 dataset, and supporting evidence from the literature. The individual expert assessments were aggregated to obtain the final criterion scores used to construct the decision matrix for the TOPSIS analysis. These values served as the input for normalization and subsequent ranking calculations.

**Table S7.** Normalized Decision Matrix Used in the TOPSIS Analysis

| Alternative | C1 | C2 | C3 | C4 | C5 |
| --- | --- | --- | --- | --- | --- |
| A1 (Teletherapy) | 0.5424 | 0.5449 | 0.5744 | 0.6032 | 0.5771 |
| A2 (In-Person Counseling) | 0.5995 | 0.4982 | 0.5026 | 0.5457 | 0.5451 |
| A3 (Blended Care) | 0.6423 | 0.6228 | 0.6175 | 0.5888 | 0.6412 |

The decision matrix was normalized using vector normalization to eliminate differences in measurement scales among the evaluation criteria. Each element was divided by the square root of the sum of squared values within the corresponding criterion. The resulting normalized decision matrix served as the basis for constructing the weighted normalized decision matrix in the subsequent TOPSIS step.

**Table S8.** Weighted Normalized Decision Matrix Used in the TOPSIS Analysis

| Alternative | C1 | C2 | C3 | C4 | C5 |
| --- | --- | --- | --- | --- | --- |
| A1 (Teletherapy) | 0.2629 | 0.1236 | 0.0822 | 0.0536 | 0.0327 |
| A2 (In-Person Counseling) | 0.2906 | 0.1130 | 0.0719 | 0.0485 | 0.0308 |
| A3 (Blended Care) | 0.3113 | 0.1413 | 0.0884 | 0.0523 | 0.0363 |

The weighted normalized decision matrix was obtained by multiplying each element of the normalized decision matrix by its corresponding AHP-derived criterion weight. This procedure incorporates both the relative importance of the evaluation criteria and the normalized performance of each alternative. The resulting matrix was subsequently used to determine the positive and negative ideal solutions and to calculate the separation distances and closeness coefficients.

**Table S9.** Positive and Negative Ideal Solutions Used in the TOPSIS Analysis

| Criterion | Positive Ideal Solution (A⁺) | Negative Ideal Solution (A⁻) |
| --- | --- | --- |
| C1 | 0.3113 | 0.2629 |
| C2 | 0.1413 | 0.1130 |
| C3 | 0.0884 | 0.0719 |
| C4 | 0.0536 | 0.0485 |
| C5 | 0.0363 | 0.0308 |

The positive ideal solution (A⁺) was constructed by selecting the maximum weighted normalized value for each benefit criterion, whereas the negative ideal solution (A⁻) was obtained by selecting the corresponding minimum values. Because all evaluation criteria were treated as benefit criteria, larger values represented better performance. The ideal solutions served as reference points for calculating Euclidean distances in the subsequent TOPSIS step.

**Table S10.** Separation Distances and Relative Closeness Coefficients of the Evaluated Alternatives

| Alternative | Distance to Positive Ideal (S⁺) | Distance to Negative Ideal (S⁻) | Closeness Coefficient (CCᵢ) | Rank |
| --- | --- | --- | --- | --- |
| A1 (Teletherapy) | 0.0563 | 0.0178 | 0.240 | 3 |
| A2 (In-Person Counseling) | 0.0372 | 0.0250 | 0.402 | 2 |
| A3 (Blended Care) | 0.0013 | 0.0592 | 0.979 | 1 |

The Euclidean separation distances from the positive ideal solution (S⁺) and the negative ideal solution (S⁻) were calculated using the weighted normalized decision matrix. The relative closeness coefficient (CCᵢ) for each alternative was then computed as $CC_{i}=S_{i}^{-}/(S_{i}^{+}+S_{i}^{-})$. The results indicate that the blended care model (A3) achieved the highest closeness coefficient (0.979), followed by in-person counseling (A2, 0.402) and teletherapy (A1, 0.240). These results are consistent with the rankings reported in the main manuscript and demonstrate that the blended care model is the preferred alternative within the proposed AHP–TOPSIS decision support framework.
